# A Large-Scale Open Motion Dataset (KFall) and Benchmark Algorithms for Detecting Pre-impact Fall of the Elderly Using Wearable Inertial Sensors

**DOI:** 10.3389/fnagi.2021.692865

**Published:** 2021-07-16

**Authors:** Xiaoqun Yu, Jaehyuk Jang, Shuping Xiong

**Affiliations:** Department of Industrial and Systems Engineering, College of Engineering, Korea Advanced Institute of Science and Technology (KAIST), Daejeon, South Korea

**Keywords:** pre-impact fall, fall detection, public dataset, wearable sensor, algorithm development

## Abstract

Research on pre-impact fall detection with wearable inertial sensors (detecting fall accidents prior to body-ground impacts) has grown rapidly in the past decade due to its great potential for developing an on-demand fall-related injury prevention system. However, most researchers use their own datasets to develop fall detection algorithms and rarely make these datasets publicly available, which poses a challenge to fairly evaluate the performance of different algorithms on a common basis. Even though some open datasets have been established recently, most of them are impractical for pre-impact fall detection due to the lack of temporal labels for fall time and limited types of motions. In order to overcome these limitations, in this study, we proposed and publicly provided a large-scale motion dataset called “KFall,” which was developed from 32 Korean participants while wearing an inertial sensor on the low back and performing 21 types of activities of daily living and 15 types of simulated falls. In addition, ready-to-use temporal labels of the fall time based on synchronized motion videos were published along with the dataset. Those enhancements make KFall the first public dataset suitable for pre-impact fall detection, not just for post-fall detection. Importantly, we have also developed three different types of latest algorithms (threshold based, support-vector machine, and deep learning), using the KFall dataset for pre-impact fall detection so that researchers and practitioners can flexibly choose the corresponding algorithm. Deep learning algorithm achieved both high overall accuracy and balanced sensitivity (99.32%) and specificity (99.01%) for pre-impact fall detection. Support vector machine also demonstrated a good performance with a sensitivity of 99.77% and specificity of 94.87%. However, the threshold-based algorithm showed relatively poor results, especially the specificity (83.43%) was much lower than the sensitivity (95.50%). The performance of these algorithms could be regarded as a benchmark for further development of better algorithms with this new dataset. This large-scale motion dataset and benchmark algorithms could provide researchers and practitioners with valuable data and references to develop new technologies and strategies for pre-impact fall detection and proactive injury prevention for the elderly.

## Introduction

The safety and health of old people have increasingly drawn attention due to accelerated global population aging. Falling is a serious problem faced by our society as 28–35% of the population aged 65 or older suffer at least one fall per year (Organization et al., 2008), and 20–30% of fall accidents lead to mild to severe injuries or even death (Lord et al., 2007). In order to mitigate the serious consequences of falls, multiple studies have been conducted to develop fall detection systems.

Based on the types of sensors being used, fall detection systems can be divided into context-aware systems and wearable systems. Context-aware systems mainly rely on ambient sensors, such as radar and floor sensors as well as vision-based devices (Igual et al., 2013). One fundamental disadvantage of such systems is that they are restricted to indoor use, so they cannot detect the fall anywhere and anytime. In fact, up to 50% of the falls happen outside home premises (Lord et al., 2007). Over the past decade, wearable inertial sensor-based fall detection systems have gained tremendous popularity among researchers because they offer high portability (no space constraints), accurate motion sensing, and low cost (Micucci et al., 2017). Therefore, this study particularly focuses on wearable inertial sensors. Generally, there are two main directions for the development of wearable inertial sensor-based fall detection systems. The majority of existing studies focus on post-fall detection, which is designed to rapidly detect fall events and initiate medical alarms timely to reduce the frequency and severity of long lies (Aziz et al., 2017). However, this approach has an inherent drawback, that is, it cannot prevent fall-induced injuries since fall impacts have already occurred. Another branch of studies targets pre-impact fall detection, which aims to detect the fall during the falling period but before body-ground impact. Therefore, it could activate on-demand fall protection systems, such as wearable airbags, to prevent injuries caused by the fall impact (Hu and Qu, 2016). This method provides a more fundamental solution for the elderly for fall injury prevention. However, it is also more challenging than post-fall detection because the sensor signal of body-ground impact moment, which includes most differentiated information (usually with peak acceleration and angular velocity), cannot be seen by algorithms.

In recent years, researchers have begun to shift their focus from post-fall detection to pre-impact fall detection and shed some light on this topic. Jung et al. (2020) developed a threshold-based algorithm, which combined multiple thresholds (magnitude of acceleration, magnitude of angular velocity, and vertical angle) based on inertial sensors for pre-impact fall detection and achieved 100% sensitivity and 97.54% specificity with an average lead time of 280 ms. This algorithm was developed based on their own simulated dataset with six types of falls and 14 types of activities of daily living (ADLs) by 30 young subjects. Another research conducted by Kim et al. (2019) applied seven machine learning algorithms and two deep learning algorithms to detect pre-impact fall, using accelerometers, and most of the models achieved ≥98% sensitivity and specificity. Similarly, those algorithms were based on their own dataset with 10 types of falls and 14 types of ADLs by 12 subjects. Quite recently, one group of researchers has proposed a multisource CNN ensemble framework for pre-impact fall detection based on the data from four pressure sensors, one acceleration sensor, and one gyro sensor (Wang et al., 2020). Ten subjects participated in their experiment, and each subject performed four types of falls and five types of ADLs. This deep learning architecture also reached high accuracy of 99.30%, with an average lead time of 350 ms. Even though the reported results were impressive, earlier studies only showed good performances of the developed algorithms on their relatively small datasets (small number of human subjects and limited types of motions), and they rarely made those datasets publicly available. This poses a challenge to fairly evaluate the performance of different algorithms on a common basis and their generalizability to different datasets. A few preliminary studies showed that algorithms based on a specific database with good performance had poor external validity on other databases (Sabatini et al., 2015; Jung et al., 2020). For instance, when Jung et al. (2020) applied their thresholds to the SisFall dataset (Sucerquia et al., 2017), both sensitivity and specificity dropped considerably by 4 and 7%, respectively. Similarly, Bourke et al. (2008) proposed an algorithm, using the vertical velocity of the trunk as the threshold and achieved 100% sensitivity and specificity on a dataset, which was built from five subjects with four types of falls and six types of ADLs. However, the same threshold with optimized value only yielded 80% sensitivity on a comparatively larger dataset with five types of falls and seven types of ADLs acquired from 25 subjects (Sabatini et al., 2015). The lack of public datasets also makes it hard to objectively compare newly developed algorithms (Noury et al., 2008). This situation thus hinders the technology advancement for pre-impact fall detection, which is expected to protect the elderly from fall injuries in a proactive way.

Some public fall databases, such as SisFall, tFall, MobiFall, and FallAllD, have been established recently. However, they are only appropriate for post-fall detection rather than pre-impact fall detection. The details will be discussed in the next section. To overcome the aforementioned limitations, in this study, we proposed and publicly provided a large-scale motion dataset called KFall. This dataset is expected to be the first public dataset suitable for pre-impact fall detection, not just for post-fall detection. We also developed three benchmark algorithms, using this new dataset, which allows researchers to fairly compare their new algorithms for pre-impact fall detection. This large-scale motion dataset and benchmark algorithms could provide researchers and practitioners with valuable data and reference to develop new technologies and strategies for pre-impact fall detection and injury prevention for the elderly.

## Related Public Fall Datasets

As mentioned in the introduction, due to the limitations of context-aware systems, the review of public fall datasets emphasized on wearable inertial sensors and was carried out through five major electronic databases (Scopus, ScienceDirect, IEEE Explorer, Web of Science, and Google Scholar). Two basic inclusion criteria were utilized for refining the search results: (1) datasets should be fully open to the public and published in English; (2) there should be at least 10 subjects in the datasets. In addition, a recent review paper, which performed a comprehensive analysis of public datasets for wearable fall detection systems, was also referred (Casilari et al., 2017a). The same group of authors has reviewed public datasets again very recently and applied CNN to those datasets for fall detection (Casilari et al., 2020). Cross-check was implemented to prevent missing any important references for this study. In the end, our search yielded 16 representative datasets (Table 1).

**Table 1:** Wearable inertial sensor-based public datasets for fall detection.

**Public dataset**	**Types of ADLs/falls**	**Subjects No**.	**Sensor data type**	**Temporal labels for the fall time**
DLR Frank et al. (2010)	15/1	19	^§^A, G, M	No
tFall Medrano et al. (2014)	Not typified/8	10	A	No
MobiFall Vavoulas et al. (2014)	9/4	24	A, G, O	No
Cogent labs Ojetola et al. (2015)	8/6	42	A, G	No
TST fall Gasparrini et al. (2015)	4/4	11	A	No
MobiAct Vavoulas et al. (2016)	9/4	57	A, G, O	No
Erciyes University Özdemir (2016)	16/20	14	^§^A, G, M	No
UMAFall Casilari et al. (2017b)	8/3	17	^§^A, G, M	No
SisFall Sucerquia et al. (2017)	19/15	38	A, G	No
UniMiB SHAR Micucci et al. (2017)	9/8	30	A	No
IMUFD Aziz et al. (2017)	8/7	10	^§^A, G, M	No
CMDFALL Tran et al. (2018)	12/8	50	A	No
CGU-BES Wang et al. (2018)	8/4	15	A, G	No
DU-MD Saha et al. (2018)	8/2	10	A	No
UP-Fall Martínez-Villaseñor et al. (2019)	6/5	17	A, G	No
FallAllD Saleh et al. (2021)	^*^44/35	15	^§^A, G, M, B	No
KFall (Our dataset)	21/15	32	A, G, O	Yes

*A, accelerometer; G, gyroscope; O, orientation measurement; M, magnetometer; B, barometer*.

§ *Complex sensor fusion algorithm should be further applied to obtain the orientation measurement*.

* *For the same type of a fall, the authors considered all possible directions (left, right, forward, backward) under two conditions (with and without recovery); 12 ADLs were hand motions, and they separated one cyclic ADL into two, such as sit down and stand up*.

As illustrated in Table 1, no dataset provides temporal labels for the fall time, which annotates the fall onset moment (when a fall begins) and the fall impact moment (when the body hits the ground) in the sensor data sequence. Lack of temporal labels for the fall time will not influence the development of algorithms for post-fall detection, since sensor data in the fall impact moment has very distinguishable patterns, which are usually with a peak value of acceleration and angular velocity. However, for pre-impact fall detection, it is important to detect the fall during the body descending period but before the moment of body-ground impact. Because of this, the algorithm for pre-impact fall detection should learn to recognize the difference of sensor data between the non-falling period and the falling period based on the known dataset. Therefore, the falling period of the sensor data, which starts from the fall onset moment and ends at the fall impact moment, should be labeled out. Unlike the fall impact moment, without temporal labels or synchronized video clips published together with motion datasets, it is almost impossible to determine the fall onset moment merely by referring to the sensor data since there is no significant signal change from preceding normal activities to the start of falls. Even though the UP-Fall dataset, the CMDFALL dataset, and the TST Fall dataset also released synchronized video references, they were at a low frequency of 18 Hz, 20 Hz, and 30 Hz, respectively. Since the entire duration of common falls is very short, with an average interval of 746 ms (Tao and Yun, 2017), such low frequency would introduce high errors when labeling the fall onset and impact moments.

Another common drawback of publicly available datasets is that most of them include limited types of falls and ADLs (≤10, e.g., DLR, tFall, MobiFall, Cogent Labs, TST Fall, MobiAct, UMAFall, UniMiB SHAR, IMUFD, CMDFALL, CGU-BES, DU-MD, and UP-Fall) to represent complex real-life scenarios. The number of human subjects used to build the dataset is also relatively small (≤20, e.g., DLR, tFall, TST Fall, Ericiyes University, UMAFall, IMUFD, CGU-BES, DU-MD, UP-Fall, and FallAllD). In addition, we also noticed that orientation data, which represent rich information of human motion (Incel, 2015), were frequently missing among the published datasets except MobiFall and MobiAct.

## Kfall Dataset Construction

We set four main requirements when designing the KFall dataset in order to complement the deficiencies of existing public datasets. (1) High-frequency synchronized video clips should be captured for labeling the fall time of sensor data; (2) the dataset should include various types of falls and ADLs with a sufficient number of subjects; (3) sensor orientation measurement should also be provided, which allows more flexibility to the interested researchers when designing their algorithms; and (4) the sampling frequency and measurement range of the inertial sensor should be sufficient (Saleh et al., 2021).

### Data Acquisition System and Experimental Setup

In order to record the sensor data together with the synchronized high-frequency video clips, a custom data acquisition system was designed. This system can be easily replicated since all the components are available from the market at affordable prices. A nine-axis inertial sensor (LPMS-B2, LP-RESEARCH Inc., Tokyo, Japan), which includes a three-axis accelerometer (±16 G), a three-axis gyroscope (±2,000°/s), and a three-axis magnetometer (±16 G), was used for collecting motion data. Orientation measurement (Euler angle: roll, pitch, and yaw) provided by the manufacturer is the integration of angular velocity and further modified, using an extended Kalman filter by combining the information from the accelerometer and the magnetometer (Petersen, 2020). The sensor was configured at a frequency of 100 Hz, which was consistent with many studies for pre-impact fall detection (Zhao et al., 2012; Wu et al., 2019). The sensor data were transmitted through Bluetooth Dongle, which was connected to Raspberry Pi 4 (4 GB) as the host PC. As for synchronous video capture, a Raspberry Pi HQ camera mounted with 6-mm 3MP Wide Angle Lens, was used at the maximum FPS of 90. Data acquisition of this research was implemented on a self-developed GUI program, running in Raspberry Pi 4 in which data synchronization between the sensor and the camera was handled by the multiprocessing technique in the Python language.

**Figure 1:**
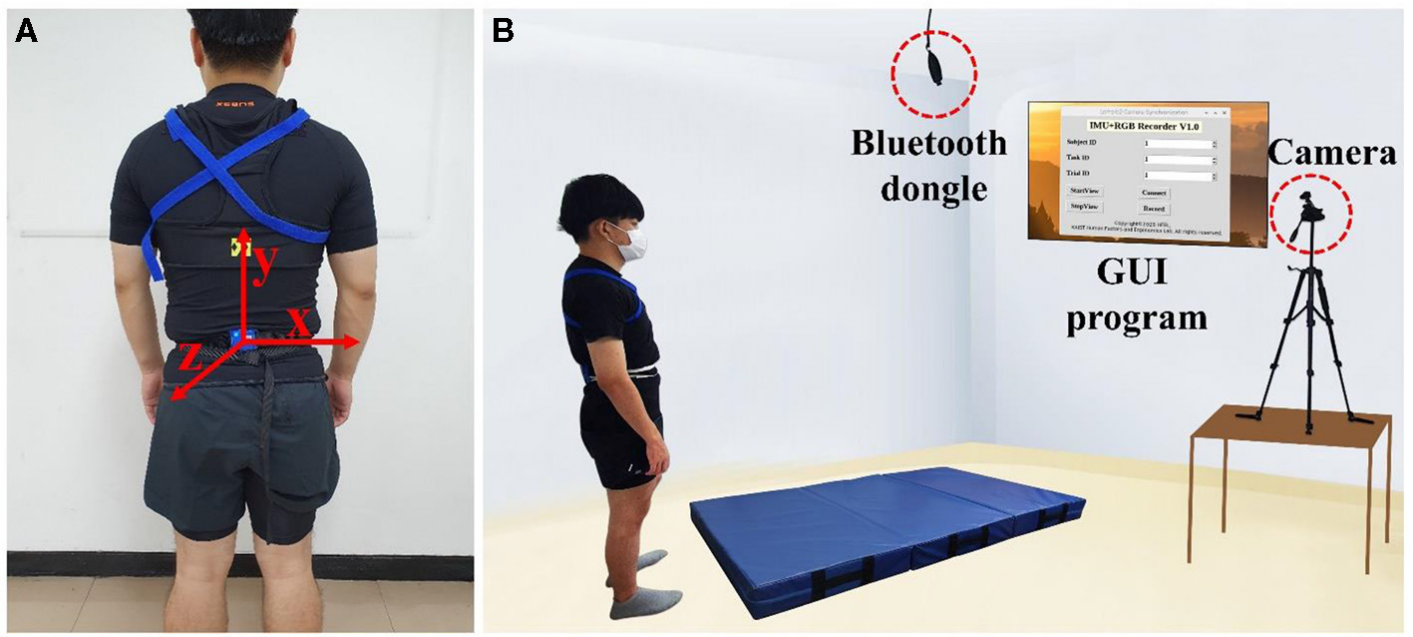
**(A)** Inertial sensor location and 3D coordinate system; **(B)** experimental setup.

The inertial sensor was attached to the low back of each subject (see Figure 1A), which was used by many researchers for fall detection (Kwolek and Kepski, 2014; Özdemir, 2016). In order to capture in-depth information of human motion, which is critical to judge the fall onset moment, the camera was set in the front side of the main experiment area rather than directly ahead of it. The whole experimental setup is shown in Figure 1B.

### Participants and Experimental Protocol

This dataset was generated from 32 young Korean males (age: 24.9 ± 3.7 years; height: 174.0 ± 6.3 cm; weight: 69.3 ± 9.5 kg). All of them were healthy and independent, and none of them reported a recent history of musculoskeletal disorder, which could affect their mobility. Every participant signed informed consent for the experimental protocol, which was approved by KAIST Institutional Review Board (IRB No: KH2020-068).

The experimental tasks in our dataset were majorly formulated from the existing public datasets (Casilari et al., 2017a). Since the SisFall dataset has the largest type of falls and ADLs and motions in other datasets were usually a subset of it, the majority of motions in our dataset were directly adopted from SisFall. All types of falls and ADLs in the SisFall dataset were chosen based on a large-scale survey among 15 independent elderly people and 17 retirement homes. For the ADLs, SisFall covers from simple daily movements (such as walking, sit to stand) to high-dynamic activities (jogging, jumping) and even near-fall scenarios (such as stumbling during walking, collapsing to a chair). As for the falls, we can divide them into three categories based on preceding activities: a fall from walking (such as caused by a slip, a trip), a fall from sitting (such as caused by fainting, trying to get up), and a fall from standing (such as trying to sit down). All the fall activities in the SisFall dataset include preceding ADLs, which are closer to the real-world falls. Another reason for choosing SisFall as our major reference is that it also provides instruction videos of each ADL and falls so that we can easily reproduce those motions.

Aside from the activities adopted from SisFall, we further added four common static activities, including sitting on a chair, sitting on a sofa, standing, and lying. Those static motions could be used as calibration postures for some fall detection algorithms (Yu et al., 2017). Considering the practical use of this dataset for the elderly population in Korea, two frequently observed ADLs in daily life of older Korean were also newly introduced. They are sitting to the ground and getting up (usually happened in restaurants), and sitting to a sofa with inclining to the back support and getting up (usually happened at home). For these reasons and the data from the Korean participants, we named our dataset as KFall. All fall activities in the KFall dataset are the same as the SisFall. While for several specific ADLs included in SisFall, we made some modifications to avoid duplication or make them more natural. For the two motions, which are sitting in a low height chair and getting up slowly or quickly, we used the motion of sitting to the ground and getting up with normal speed (D05) to replace them since they have similar motion patterns. Likewise, with regard to two motions which are standing, slowly bending with or without bending knees and getting up, tying shoelaces (D02) with or without bending at knees, and getting up are the substitutes. We also removed two ADLs considered in the SisFall dataset. One is to rotate the body when lying in a bed since it usually occurs during sleep. Another one is to get into and out of a car due to the facility constraint. Finally, 21 types of ADLs and 15 types of simulated falls were included in the KFall dataset (Table 2). Except for the static tasks (D01, D11, D12, and D17), which required only one trial, all other tasks were designed for five trials. During the experiment, for the ADLs, the subjects were instructed to perform them based on their daily habits to make those motions as natural as possible. While for the fall activities, since the young subjects usually do not have fall experience, instruction videos from the SisFall and on-site demos by experimenters were provided if necessary. To ensure the subject safety, all the fall activities were performed on a 15-cm-thick mattress. After the experiment, incomplete data caused by Bluetooth signal disconnections or synchronization errors were removed. Finally, KFall contains a total of 5,075 motion files, including 2,729 ADL motions and 2,346 fall motions.

**Table 2:** Experimental tasks of 21 types of ADLs and 15 types of falls.

**Task ID**	**Activity**	**Trials**
D01	Stand for 30 s	1
D02	Stand, slowly bendthe back with or without bending at knees, tie shoe lace, and get up	5
D03	Pick up an object from the floor	5
D04	Gently jump (try to reach an object)	5
D05	Stand, sit to the ground, wait a moment, and get up with normal speed	5
D06	Walk normally with turn (4 m)	5
D07	Walk quickly with turn (4 m)	5
D08	Jog normally with turn (4 m)	5
D09	Jog quickly with turn (4 m)	5
D10	Stumble while walking	5
D11	Sit on a chair for 30 s	1
D12	Sit on the sofa (back is inclined to the support) for 30 s	1
D13	Sit down to a chair normally, and get up from a chair normally	5
D14	Sit down to a chair quickly, and get up from a chair quickly	5
D15	Sit a moment, trying to get up, and collapse into a chair	5
D16	Stand, sit on the sofa (back is inclined to the support), and get up normally	5
D17	Lie on the bed for 30 s	1
D18	Sit a moment, lie down to the bed normally, and get up normally	5
D19	Sit a moment, lie down to the bed quickly, and get up quickly	5
D20	Walk upstairs and downstairs normally (five steps)	5
D21	Walk upstairs and downstairs quickly (five steps)	5
F01	Forward fall when trying to sit down	5
F02	Backward fall when trying to sit down	5
F03	Lateral fall when trying to sit down	5
F04	Forward fall when trying to get up	5
F05	Lateral fall when trying to get up	5
F06	Forward fall while sitting, caused by fainting	5
F07	Lateral fall while sitting, caused by fainting	5
F08	Backward fall while sitting, caused by fainting	5
F09	Vertical (forward) fall while walking caused by fainting	5
F10	Fall while walking, use of hands to dampen fall, caused by fainting	5
F11	Forward fall while walking caused by a trip	5
F12	Forward fall while jogging caused by a trip	5
F13	Forward fall while walking caused by a slip	5
F14	Lateral fall while walking caused by a slip	5
F15	Backward fall while walking caused by a slip	5

### Data Labeling for Fall Time

**Figure 2:**
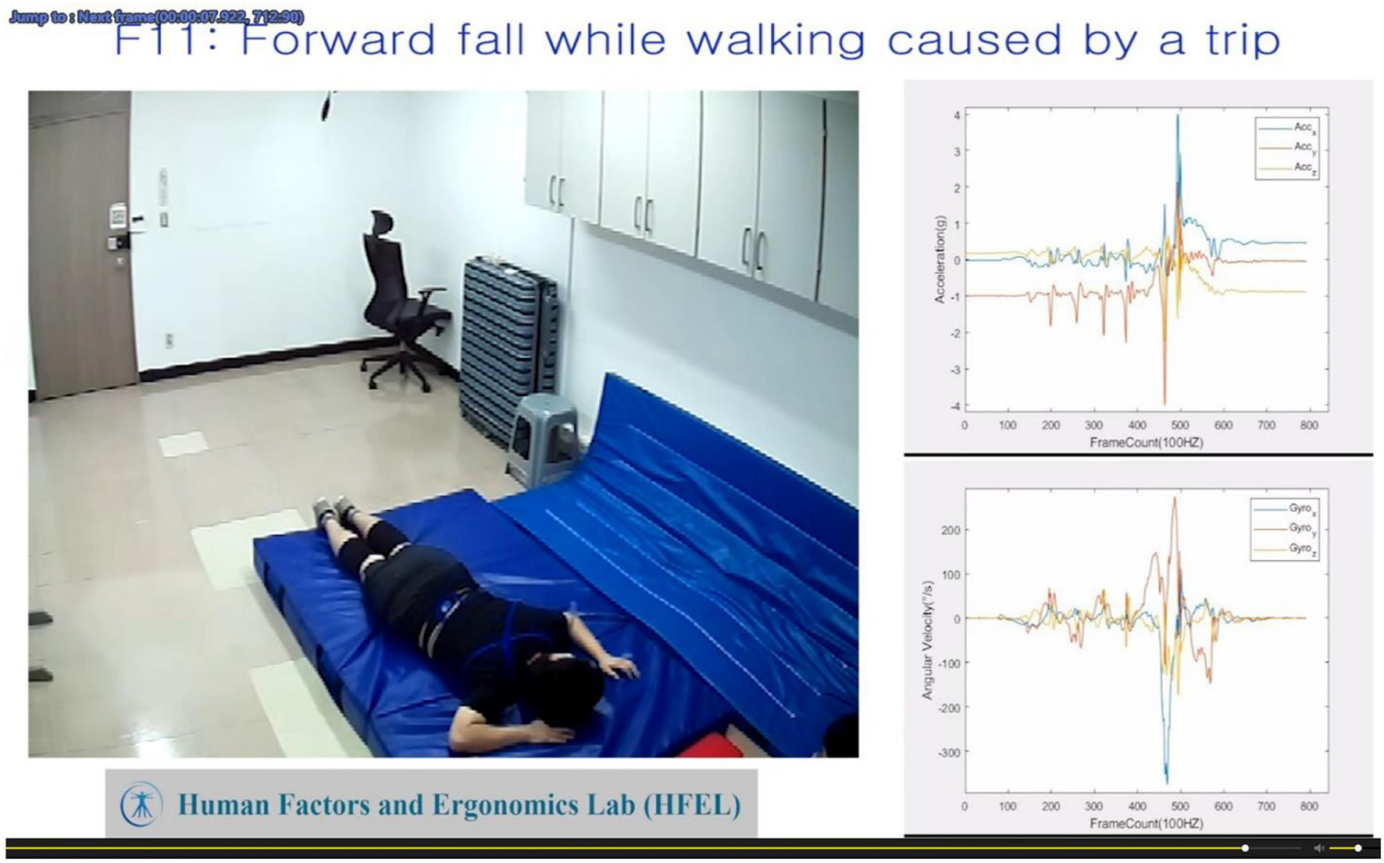
Integrated motion video for the fall time labeling. Left: synchronized fall motion video from the camera; right: sensor data video converted from readings of the inertial sensor (acceleration and angular velocity).

Since the existing public fall datasets lack synchronized video references on fall activities, it is very difficult to reliably label the fall onset moment solely based on the sensor data for pre-impact detection. On the other hand, because naked eyes cannot recognize subtle motions in the graphics, only referring to the video will also introduce large errors in the labeling process. Therefore, we propose a new method that combines information from both sensor and video data to reduce the labeling error. We firstly converted the sensor data in a csv format into a video format (avi, 100 Hz) and further integrated with a fall motion video (90 Hz) from a Raspberry HQ camera as a whole video, which was played at 90 Hz (see Figure 2). The video from the sensor data and the camera maintained the same frequency as the original data format to avoid time distortion. Secondly, based on the integrated video, we played synchronized fall motion and sensor data video frame by frame to accurately label the fall onset and fall impact moments.

**Figure 3:**
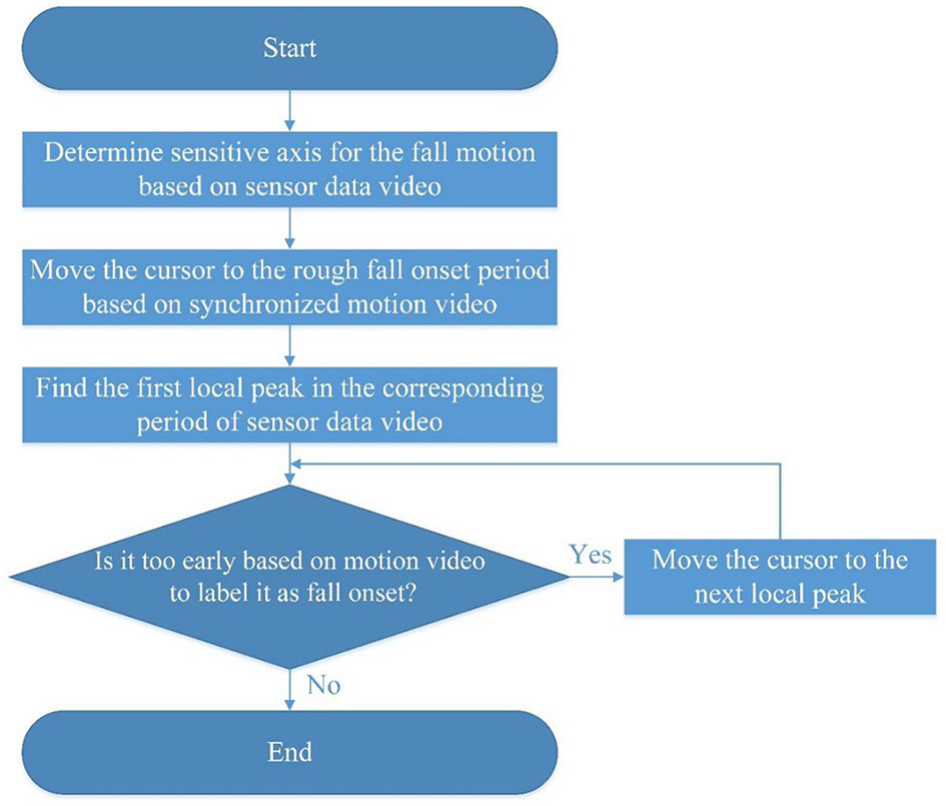
A flowchart for labeling the fall onset moment in sensor data.

**Figure 4:**
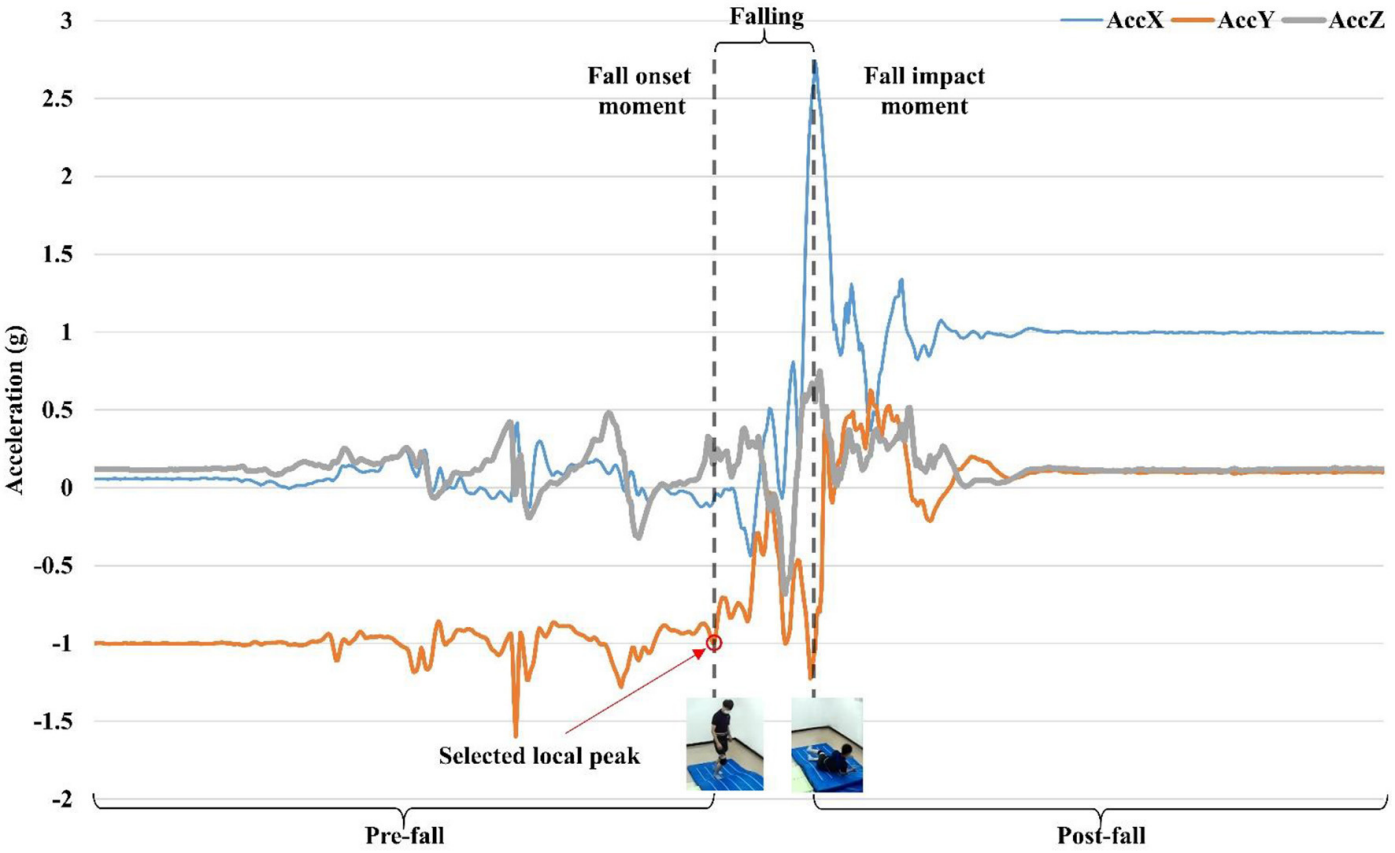
Illustration of fall time labeling during a fall event based on the integrated motion video.

The fall impact moment can be easily determined since body-ground impact and an acceleration peak are obvious in the integrated video. Whereas, for the fall onset moment, there is a less obvious motion pattern, and it is hard to define it quantitatively. For this reason, we have introduced a semiautomatic method for labeling the fall onset moment after a comprehensive review of the integrated fall videos (see Figure 3). Since the fall is preceded by dynamic movements (e.g., walking, getting up, and sitting down), the y-axis of acceleration is usually considered as a sensitive axis. This is because the acceleration on the y-axis shows the most obvious pattern during falling. Falls usually have significant motion changes in the gravity direction, which can be detected by the y-axis acceleration. However, for the falls caused by fainting during sitting, they are less dynamic and usually without obvious acceleration change at the beginning of falling. Therefore, the y-axis of acceleration could not be regarded as the sensitive axis. In such cases, the x-axis or z-axis of the angular velocity can be considered as the sensitive axis because there are more significant changes during rotational movements along the sagittal plane (a forward/backward fall) or the frontal plane (a lateral fall). Those body motion changes during different fall activities (Bourke et al., 2010) reflect local peaks in sensor signals along the sensitive axis. Based on the synchronized fall motion video, we can quickly move the cursor along the timeline to the rough period of the fall onset. Then we evaluated local peaks in the corresponding period of the sensor data video one by one. Those local peaks could be regarded as potential candidates of the fall onset moment. Choosing the local peak among the candidates involves some subjective judgments of the evaluator. Since the proposed semiautomatic labeling method achieved a high degree of consistency in labeling the fall onset moments between the two independent evaluators in the pilot test, the KFall dataset was labeled by an experienced evaluator for time efficiency. One representative case of labeling is illustrated in Figure 4, which is a forward fall during walking caused by a slip. Based on the fall onset and the fall impact moments, the fall event could be further divided into three phases: pre-fall, falling, and post-fall phases. Since our major focus is to detect the fall before body-ground impact (a pre-impact fall), the post-fall phase is not considered for the following algorithm development.

**Figure 5:**
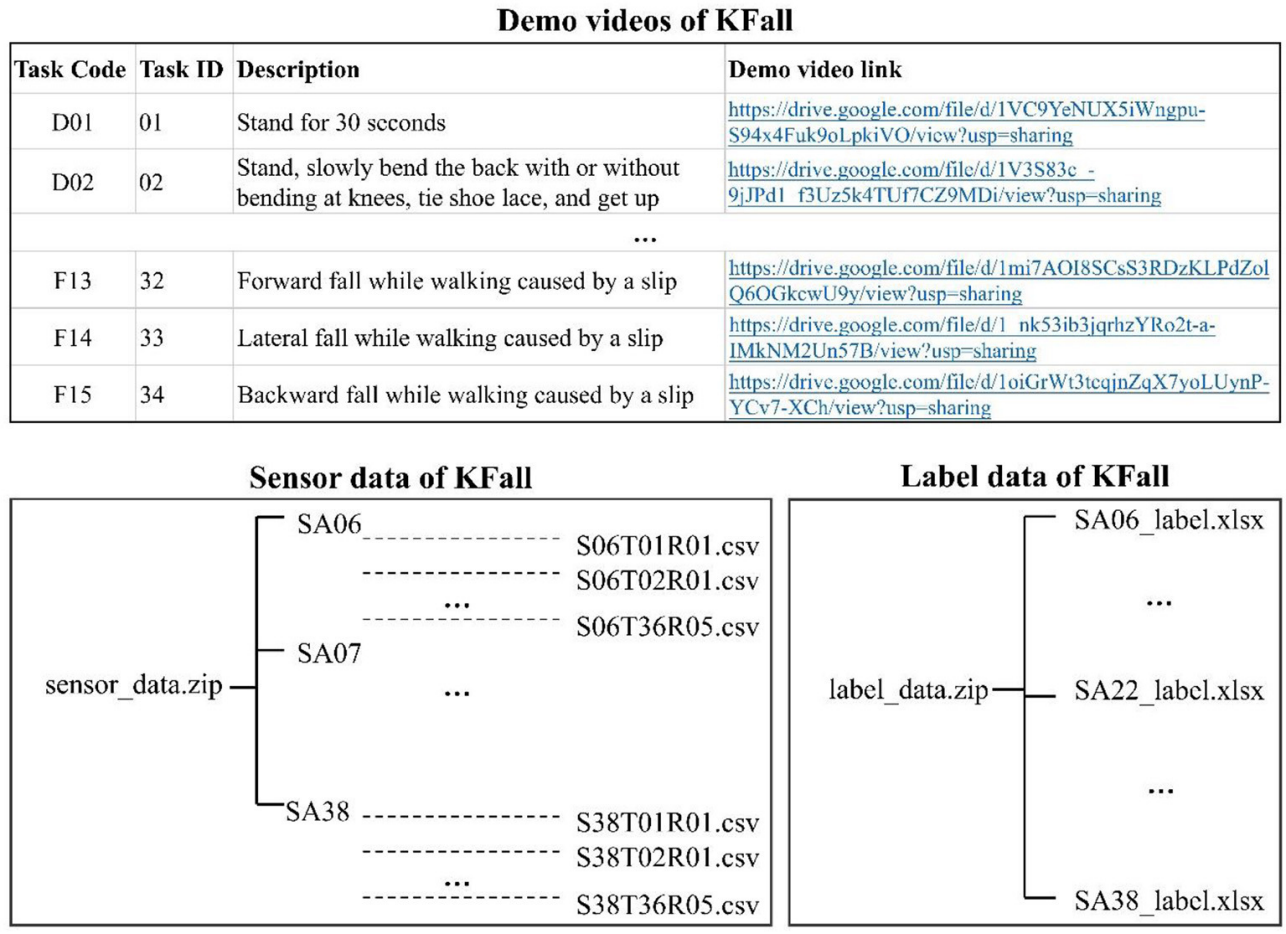
Organization of the KFall dataset provided in the website.

All the sensor data, labels of fall trials, and demo videos are publicly available from the Google site: https://sites.google.com/view/kfalldataset. The detailed data organization is summarized in Figure 5. For each motion file (csv), it contains 11 columns, which are TimeStamp(s), FrameCounter, acceleration (unit: g), angular velocity (unit: °/s), and Euler angle (°) along three axes. Each label file (xlsx) includes temporal labels for the fall time of all the fall trials from each subject, and it has six columns: task code (task ID), description, trial ID, fall onset frame, and fall impact frame in the sensor data. All the demo videos can be accessed from the attached links.

## Benchmark Algorithms for Pre-Impact Fall Detection

Based on our newly developed motion dataset KFall, we further developed and tested three different types of algorithms for pre-impact fall detection. Those algorithms cover three major distinct categories in the literature: (i) threshold-based (ii) conventional machine learning, and (iii) deep learning algorithms.

**Figure 6:**
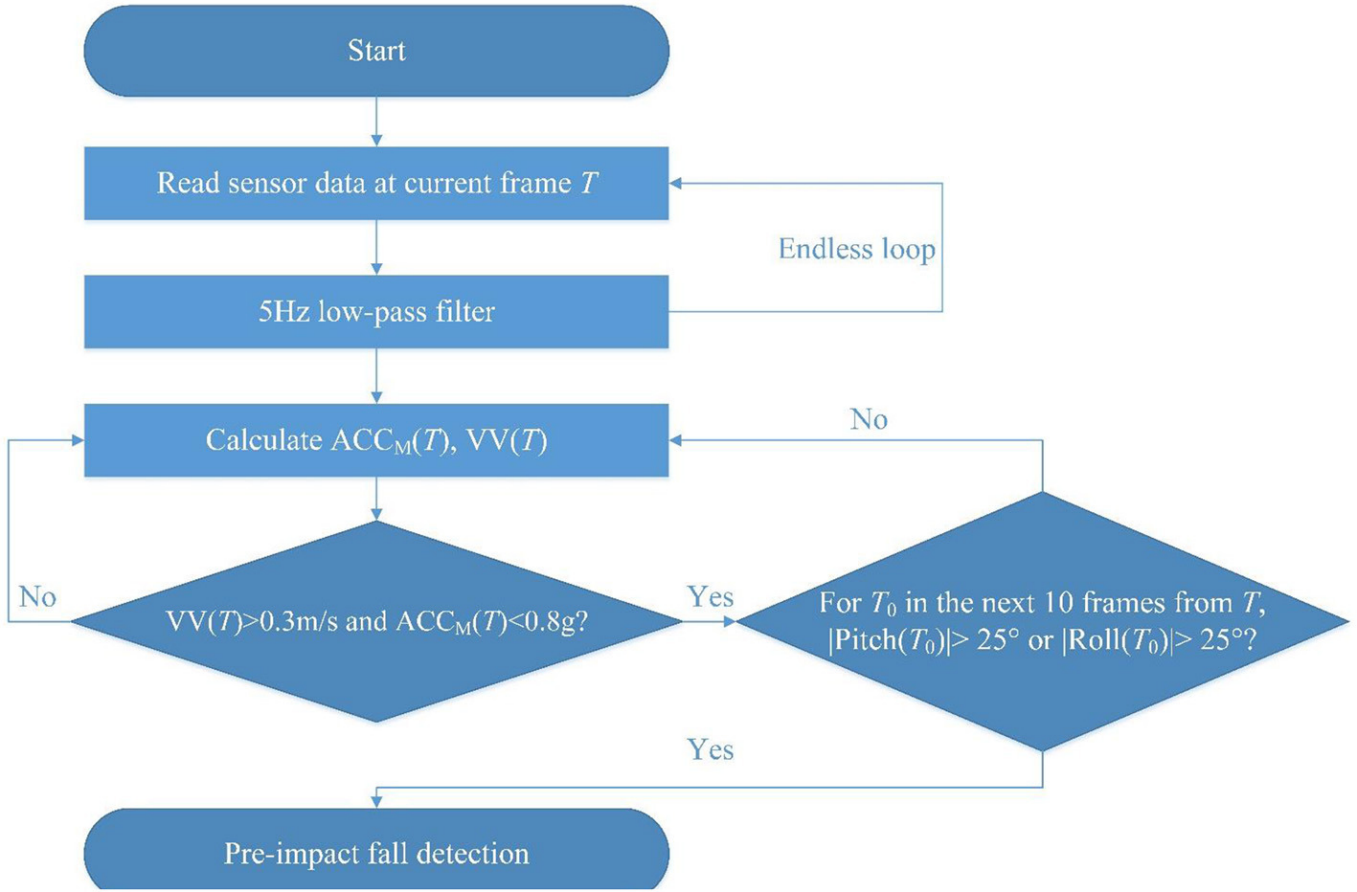
A flowchart of the threshold-based algorithm for pre-impact fall detection.

For the threshold-based algorithm, four thresholds (magnitude of acceleration, pitch angle, roll angle, and vertical velocity) were considered to detect a pre-impact fall based on recent publications (Jung et al., 2020; Kim et al., 2020). The magnitude of the acceleration is the L-2 norm of acceleration readings from three axes. Pitch and roll angles are defined as the rotations around the X-axis and Z-axis of the sensor. As for the vertical velocity, it is calculated by integrating the vertical acceleration, which is obtained by the Euler angle transformation of the three-axis acceleration data (Lee et al., 2014). The optimal threshold values were determined by the grid search method. Finally, the threshold values of the magnitude of acceleration (ACC_M_), pitch angle, roll angle, and vertical velocity (VV) were set to 0.8 g, 25°, 25°, and 0.3 m/s, respectively. Figure 6 shows the flowchart of the threshold-based algorithm for detecting pre-impact falls.

For the conventional machine learning algorithm, support vector machine (SVM) was applied in this study since it usually achieved better performance in similar tasks (Aziz et al., 2017; Qiu et al., 2018). A comprehensive set of motion features, which encompassed acceleration, angular velocity, and orientation-based information in both temporal and frequency domains, was selected (Incel, 2015). The magnitude of acceleration and angular velocity of each sliding window, with the width of 50 frames (0.5 s) were utilized for extracting features from the acceleration and angular velocity data (Aziz et al., 2014). Basic features include mean, variance, and root mean square (RMS). More advanced ones are listed as follows: (1) zero-crossing rate (ZCR): the number of samples, which is over the mean of the window; (2) absolute difference (ABSDIFF): the sum of the absolute difference between each sample and the mean of the window divided by the number of samples; (3) First 5-FFT coefficients: the first five of the fast Fourier transform (FFT) coefficients; (4) spectral energy (SE): the sum of the squared FFT coefficients divided by the number of samples. With respect to the orientation-based features, they were calculated from the pitch, roll, and yaw angles. Likewise, mean, standard deviation, RMS, ZCR, ABSDIFF, and SE were derived from those angles. Finally, a total of 40 features were generated and further normalized as the input for the machine learning model.

**Figure 7:**
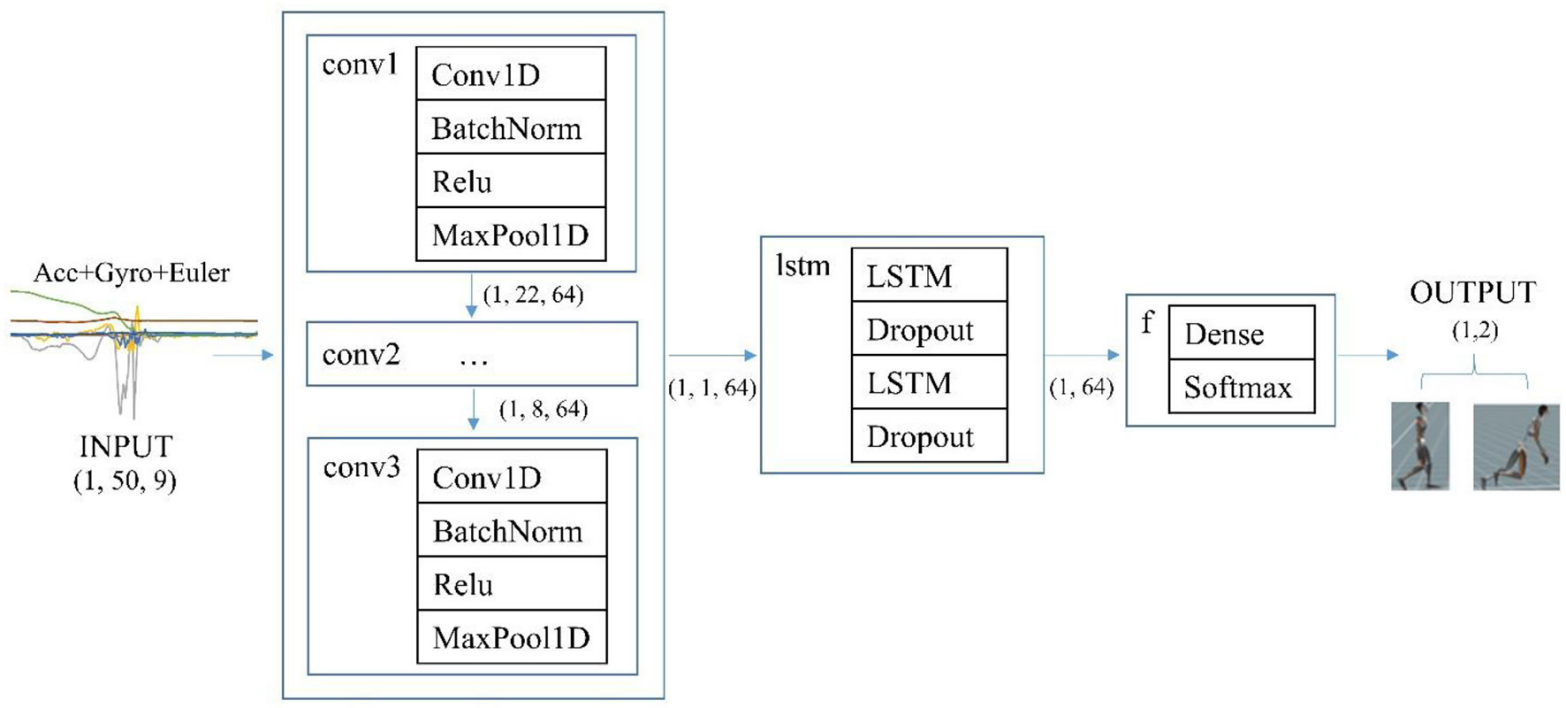
The architecture of the deep learning model (ConvLSTM).

For the deep learning algorithm, a novel hybrid architecture, integrating both convolution and long short-term memory (ConvLSTM, Figure 7) was adopted from our latest publication (Yu et al., 2020). The model consists of three sequential convolutional blocks followed by two long short-term memory (LSTM) cells with dropout operations and one fully connection layer with softmax activation. Each convolutional block contains operations of convolution, batch normalization, relu, and max pooling. The preceding convolutional layers were designed as automatic feature extractors to provide abstract representations of the sensor raw data in the feature maps. Those high-level features with short-term dependencies were further processed by the recurrent layers, which could capture the long-term temporal relationship of the motion data. Nine-dimensional sensor raw data (three-axis acceleration, three-axis angular velocity, and three Euler angles), with a window size of 50 frames (0.5 s), were the input of the ConvLSTM model.

Following the general guideline, 80% of data (26 subjects) were randomly chosen as a training set, and the rest 20% of data (six subjects) were treated for testing purposes. Sensitivity, specificity, and lead time were calculated to evaluate the performance of three different algorithms. Lead time was defined as the time interval between the fall detection moment (when a fall was detected by the algorithm) and the fall impact moment. Sufficient lead time is an important practical application requirement for algorithms to be deployed to on-demand fall protection systems (such as wearable airbags). Algorithm sensitivity and specificity were calculated by equations 1 and 2, respectively.

(1) $$\text{Senvitivity}=\frac{TP}{\text{TP}+\text{FN}}$$

(2) $$\text{Specificity}=\;\frac{\text{TN}}{\text{TN}+\text{FP}}\;$$

where TP (true positive) is the number of fall files detected as falls; FN (false negative) is the number of fall files detected as ADLs; TN (true negative) is the number of ADL files detected as ADLs; FP (false positive) is the number of ADL files detected as falls.

Table 3 shows the overall performance of three different algorithms on the testing set, which contains 444 fall files and 507 ADL files. In terms of accuracy-related measures, conventional machine learning algorithm (SVM) and deep learning algorithm (ConvLSTM) outperformed the threshold-based algorithm. Particularly, ConvLSTM achieved both high overall accuracy and balanced sensitivity (99.32%) and specificity (99.01%). SVM also had a good performance with sensitivity of 99.77% and specificity of 94.87%. However, the threshold-based algorithm showed relatively poor results, especially the specificity (83.43%) was much lower than the sensitivity (95.50%). With respect to lead time, ConvSLTM obtained the best performance with an average lead time of 403 ± 163 ms, which was slightly longer than SVM (385 ± 159 ms) but much longer than the threshold-based algorithm (333 ± 160 ms).

**Table 3:** Overall performance of three benchmark algorithms on the testing set.

**Algorithm**	**FN**	**FP**	**Sensitivity (%)**	**Specificity (%)**	**Lead time (ms)**
Threshold	20/444	84/507	95.50	83.43	333 ± 160
SVM	1/444	26/507	99.77	94.87	385 ± 159
ConvLSTM	3/444	5/507	99.32	99.01	403 ± 163

*FN, false alarm; FP, false positive*.

## Algorithm Validation on Real-World Fall Dataset (Farseeing)

In order to further evaluate the feasibility of applying trained algorithms based on our simulated fall dataset of young volunteers to detect real-world falls in the elderly, the FARSEEING dataset, currently, the largest real fall repository (Klenk et al., 2016; Chen et al., 2017), was used in this study. A total of 22 records of real-world falls are available upon request. Each fall file contains 1,200 s of data, including data signals of ADLs and falling. In this study, 15 falls were selected because they were collected from the sensors with the same location and data acquisition frequency as our KFall. Those 15 falls were collected from eight older adults (two males, six females; age: 66.9 ± 6.5 years; height: 162.2 ± 9.3 cm; weight: 74.2 ± 10.3 kg). Since half of fall samples lack angular velocity data and they all lack orientation data, in order to fully utilize this dataset, only acceleration data were used. The best model (ConvLSTM) among three benchmark algorithms was selected for the validation purposes. Without changing the network structure, it was retrained only based on the acceleration data from KFall. Since the FARSEEING dataset does not have a video reference, the fall onset moment is defined as 1 s prior to the fall impact moment as in other studies (Shi et al., 2012; Chen et al., 2017). The data before the fall onset moment were segmented as an ADL file, and the data between the fall onset moment and the fall impact moment were segmented as a fall file, resulting in 15 ADL files and 15 fall files. The data after the fall impact moment were not considered for pre-impact fall detection as we did in the KFall. The same window size of 50 frames (0.5 s) was also applied. The performance of ConvLSTM on the FARSEEING dataset is summarized in Table 4. The results showed that the ConvLSTM model achieved a sensitivity of 93.33% (= 14/15), a specificity of 73.33% (= 11/15), and an averaged lead time of 411 ms.

**Table 4:** Validation performance of ConvLSTM on the FARSEEING real-world fall dataset.

**Algorithm**	**FN**	**FP**	**Sensitivity (%)**	**Specificity (%)**	**Lead time (ms)**
ConvLSTM	1/15	4/15	93.33	73.33	411 ± 317

*FN, false alarm; FP, false positive*.

## Discussion

Pre-impact fall detection based on wearable inertial sensors is still a research problem to be solved. Most of papers only published algorithms, using their own datasets but rarely made them publicly accessible, which hinders the development of pre-impact fall detection and proactive injury prevention. So far, there is no open fall dataset suitable for pre-impact fall detection; therefore, we newly established the KFall dataset and made it publicly available for the first time. This motion dataset was developed from the 32 Korean participants while performing 21 types of ADLs and 15 types of falls. This dataset covers almost all typical daily activities and falls, and it is expected to provide researchers and practitioners with a common foundation to develop new algorithms and technologies on pre-impact fall detection and proactive injury prevention.

Compared with the existing public fall datasets (Casilari et al., 2017a; Saleh et al., 2021) in the literature, the biggest advantage of the KFall dataset is that it is constructed with the synchronized video reference and motion sensor data. This enables accurate temporal labels for the fall time and allows the dataset to be further used for pre-impact fall detection, not just post-fall detection. We also proposed a new and semiautomatic method for reliably labeling the fall onset moment by checking local peaks of the sensor data through the integrated motion and sensor data video (Figures 2, 3). Even though this method still involves some subjective judgments from human evaluators, the induced variations should be minimal because the video is at a high frame rate (90 Hz). Musci et al. (2020) conducted an interesting study for pre-impact fall detection based on the SisFall dataset. Due to the lack of video references in the SisFall dataset, the authors and their colleagues formed an expert panel to label the fall time only based on the sensor data pattern. This approach is very difficult to implement and tends to be less accurate, especially for some falls with very short intervals, such as a backward fall when trying to sit down. In order to maintain the privacy of the participants, the synchronized videos are not open to the public, whereas ready-to-use labels of each fall trial (a fall onset frame and a fall impact frame) are published together with the KFall dataset. Another strength of this dataset is that it contains a comparable number of motion types and human subjects as the three most comprehensive datasets (SisFall, Erciyes University, and FallAllD) in the literature (Table 1). It covers different physical levels of ADLs from low-activity behaviors to high-dynamics and even near-fall scenarios, and also covers from less-intensive falls (such as caused by fainting) to very dynamic falls (such as caused by a slip or a trip). This dataset is closer to the complex real-world scenarios, so it is more valuable for research and development in the field of pre-impact fall detection and proactive injury prevention.

In addition, three different types of algorithms for pre-impact fall detection were implemented based on this comprehensive motion dataset. All of them were adopted from the state-of-the-art algorithms published recently (Jung et al., 2020; Kim et al., 2020; Yu et al., 2020, 2021) and thus were representative to be the benchmarks. It was expected that the threshold-based algorithm showed poorer performance compared with machine learning algorithms (SVM and ConvLSTM) since the number of motion features considered for the threshold-based algorithm was much less than the other two algorithms. It is usually infeasible to include many thresholds for threshold-based algorithms, which would dramatically increase the searching space and introduce undermined results (e.g., one fall, which satisfies some thresholds, could be against other thresholds). With respect to machine learning algorithms, ConvLSTM had a more balanced sensitivity and specificity (99.32 and 99.01%) than SVM (99.77 and 94.87%). Compared with hand-crafted features used in SVM, the automatic features generated by well-designed deep learning neural networks had more distinguishing power of ADLs and falls (Wang et al., 2019). As for the lead time, it is a critical performance indicator for practical applications, such as on-demand airbags for fall injury prevention. In such a wearable system, a short lead time may fail to prevent fall-induced injuries since it is too short to fully inflate the airbag before the body-ground impact. In this work, both ConvLSTM (403 ± 163 ms) and SVM (385 ± 159 ms) showed a much longer lead time than the threshold-based algorithm (333 ± 160 ms), considering the very short duration of falling (~746 ms as reported earlier). This fact also indicates that the features used in ConvLSTM and SVM are more comprehensive and robust to distinguish falls at the beginning stage of falling from ADLs. Readers should be aware that, in this study, only accuracy measures (sensitivity and specificity) and lead time were considered to evaluate the benchmark algorithms. Other practical issues, such as the computational resource and battery capacity in wearable embedded devices, should be explored in the future to have a more comprehensive evaluation of the algorithms (Torti et al., 2018).

It is worth discussing the sampling frequency and location of the sensor used in our KFall dataset. Since KFall is designed for pre-impact fall detection and proactive injury prevention rather than post-fall detection, only part of the fall data can be seen by the pre-impact fall detection algorithms. Considering the short period of falling (average 746 ms, Tao and Yun, 2017) and the buffer time required for the full activation of fall protection devices, such as inflatable airbags, low-frequency sensor data may not provide sufficient motion information and fine details for accurate classification, especially for machine and deep learning algorithms, because they extract features from sliding windows of multiple frames. In a recent review paper on pre-impact fall detection (Hu and Qu, 2016), only three out of 13 studies set the sensor sampling frequency below 100 Hz, and all of these studies applied threshold-based algorithms. Threshold-based algorithms are less sensitive to the sampling frequency since their working principle is usually based on a single frame of data, not multiple frames. Even for post-fall detection, Saleh et al. (2021) found that the detection accuracy was always improved by increasing the sensor sampling frequency from 20 to 40 Hz in three different sensor locations. In this study, KFall with a sensor sampling frequency of 100 Hz achieved promising accuracy and lead time in three benchmark algorithms, which also provides some flexibility for interested readers to evaluate the performance of different algorithms if downsampling is required. Regarding the sensor location, low back was chosen for the KFall dataset due to two main reasons. First, low back has been validated as one of the best sensor positions for fall detection (Ntanasis et al., 2016; Özdemir, 2016), and many studies on pre-impact fall detection have also achieved good accuracy from this position (Shan and Yuan, 2010; Jung et al., 2020). This is understandable since the low back position is close to the center of mass of the human body. Therefore, the motion data collected from this location could represent human motion well. The second reason is related to practical applications of preventing fall-related injuries. Since a hip fracture is one of the most serious fall-related injuries, it can reduce mobility and even cause death (Lord et al., 2007; Jung et al., 2020); the sensor in this location can be easily embedded into a belt-shaped airbag for protecting the hip in real time (Shi et al., 2009; Tamura et al., 2009; Ahn et al., 2018).

There is still an ongoing debate about the effectiveness of applying algorithms trained on simulated fall datasets of the young volunteers to real-world fall detections in the elderly. Klenk et al. (2011) observed that, compared with simulated falls, real-world falls have considerably larger changes in acceleration during the falling phase. While other researchers reported similar features in acceleration signals between two types of falls, they also found that some fall phases detected from simulated falls were not detectable from real falls (Kangas et al., 2012). Bagala et al. (2012) evaluated 13 published threshold-based algorithms on an acceleration dataset with 29 real-world falls. Their results showed the average sensitivity and specificity were 57 and 83%, respectively, which were much worse than the results of detecting simulated falls. On the contrary, another group of researchers trained the SVM algorithm based on their simulated fall dataset and further tested the model on the FARSEEING real-world fall dataset (Chen et al., 2017); they achieved both high sensitivity and specificity (>95%) in detecting real-world falls. The potential reason for this conflicting result may be that the features extracted from windows in machine learning algorithms have more distinguishing power than the features extracted from discrete frames in threshold-based algorithms. However, both studies only focused on post-fall detection because they did not investigate whether the fall was detected before the body hit the ground. In this study, we also used the FARSEEING real-world fall dataset to externally validate the best trained ConvLSTM model from our simulated fall dataset (KFall) for pre-impact fall detection. The results showed high sensitivity since 14 out of the 15 real-world falls were successfully detected before body-ground impact (Table 4). However, the specificity dropped sharply compared to the performance in the simulated fall dataset, which could be understandable since only acceleration data were used to train the model due to the lack of angular velocity and orientation data in FARSEEING dataset. Our validation results demonstrated a certain potential of using the simulated dataset for real-world pre-impact fall detection. For better validation, a larger real-world fall dataset with more comprehensive motion signals (including acceleration, angular velocity, and orientation) is needed.

There are some limitations related to our current KFall dataset. First, the current KFall only contains simulated falls from young male adults due to safety concerns and practical convenience. Caution is thus needed to directly apply KFall dataset into real-world applications. Second, the current KFall dataset does not include normal ADLs from older subjects due to some practical limitations from the COVID-19 pandemic. We will further expand our KFall dataset by recruiting older and female subjects as well as evaluate false alarm rates of benchmark algorithms on the target population in the future.

## Conclusion

In this paper, we proposed and publicly provided a comprehensive motion dataset called “KFall” for pre-impact fall detection. This new dataset was developed from 32 Korean participants while performing 21 types of ADLs and 15 types of falls. The motion data contain acceleration, angular velocity, and Euler angle, which are collected from a nine-axis inertial sensor attached at the low back of each participant. Compared with the existing public datasets, the advantages of the KFall dataset are 3-fold. First of all, it covers almost all typical ADLs and falls, thus getting closer to the complex real-world scenarios. Secondly but more importantly, the KFall dataset is constructed together with a synchronized video camera at a high frame rate of 90 Hz, which makes it the first public dataset for pre-impact fall detection, not just for post-fall detection. In this process, we further introduced a practical and semiautomatic method to label the fall onset moment by integrating information from the sensor and video data. Thirdly, we also developed three different types of state-of-the-art algorithms (threshold based, a support-vector machine, and deep learning), using the KFall dataset for pre-impact fall detection. Performance of these algorithms could be regarded as benchmarks for further developing better algorithms with this new dataset. This large-scale motion dataset and benchmark algorithms could provide researchers and practitioners valuable data and references to develop new technologies and strategies for pre-impact fall detection and proactive injury prevention for the elderly.

## Data Availability Statement

The datasets presented in this study can be found in online repositories. The names of the repository/repositories and accession number(s) can be found in the article/supplementary material.

## Ethics Statement

The studies involving human participants were reviewed and approved by KAIST Institutional Review Board. The patients/participants provided their written informed consent to participate in this study.

## Author Contributions

SX conceptualized the study, obtained the funding, and reviewed the edited manuscript. XY developed the data acquisition software, developed the benchmark algorithms, and wrote the original draft. XY and JJ conducted the experiment. All authors contributed to the article and approved the submitted version.

## Conflict of Interest

The authors declare that the research was conducted in the absence of any commercial or financial relationships that could be construed as a potential conflict of interest.
